# Supplementary material for: Tight cohesion between glycolipid membranes results from balanced water–headgroup interactions
Source: Nat Commun. 2017 Apr 3;8:14899. doi: 10.1038/ncomms14899 (PMC5382269; doi:10.1038/ncomms14899)
Supplement: Supplementary Information — Supplementary Figures, Supplementary Notes and Supplementary References [file ncomms14899-s1.pdf]

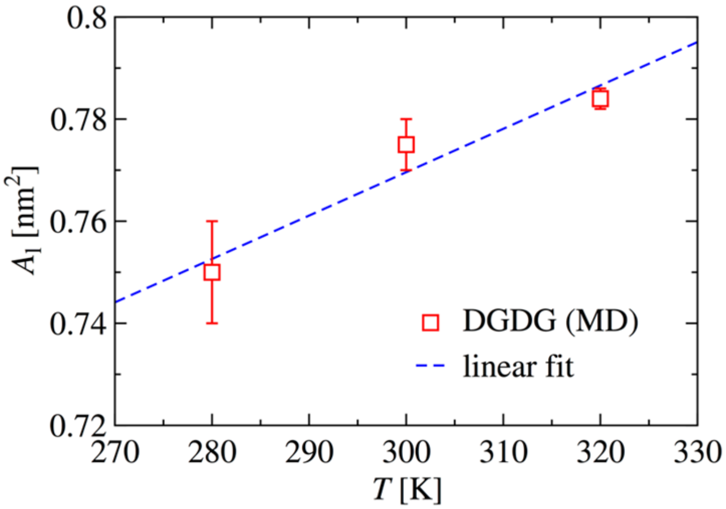

Supplementary Figure 1. **Thermal expansion of DGDG.** Temperature dependence of the area per DGDG lipid (red squares). The dashed straight line is a linear fit to the data points. See Supplementary Note 1.

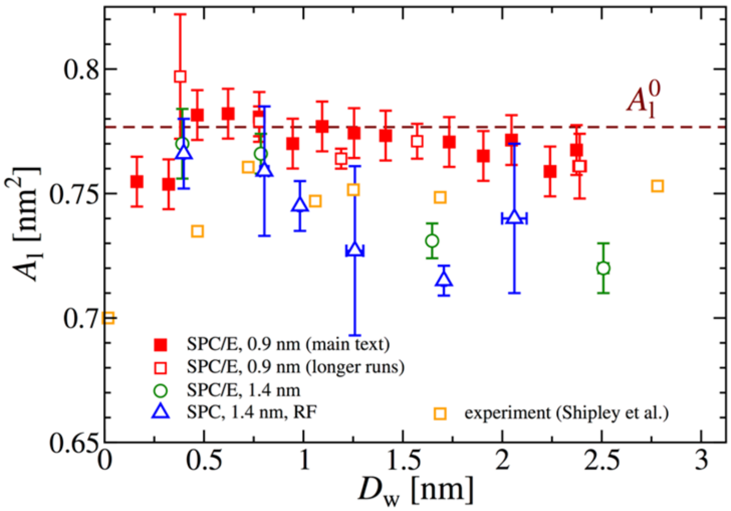

Supplementary Figure 2. **Sensitivity to the simulation parameters.** Area per DGDG lipid as a function of the water layer thickness D_W for SPC/E water, PME electrostatics, and r_{LJ} = 0.9 nm (our study), for SPC/E water, PME electrostatics, and r_{LJ} = 1.4 nm (green circles), and for SPC water, RF electrostatics, and r_{LJ} = 1.4 nm (blue triangles). See Supplementary Note 2.

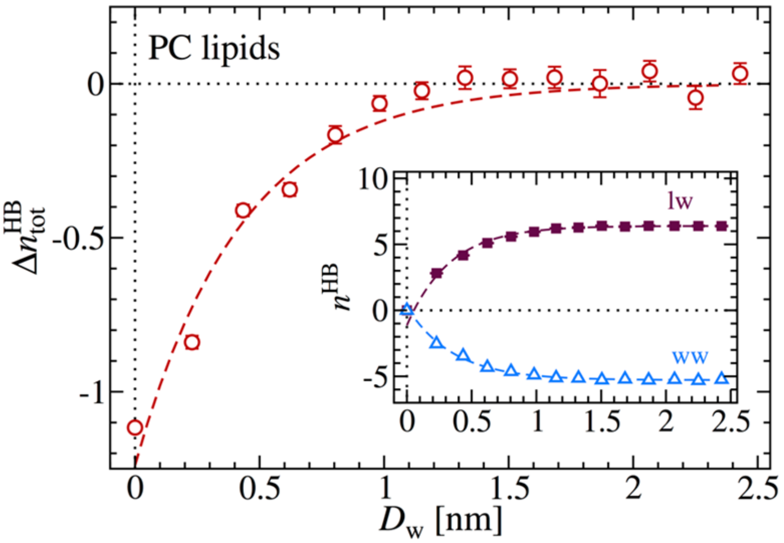

Supplementary Figure 3. **Hydrogen bond decomposition for PC lipids.** Total number of hydrogen bonds (HBs) per PC lipid as a function of the membrane separation. The dashed line indicates an exponential fit with decay length $\lambda = 0.43$ nm. Inset: Lipid–water (lw) and water–water (ww) HBs. In the plot, ww refers to the excess HB number with respect to bulk water, $n_{ww}^{HB} - n_w n_{bw}^{HB}$, see main text. Dashed lines indicate exponential fits with decay lengths $\lambda = 0.36$ nm for n_{ww}^{HB} and $\lambda = 0.33$ nm for n_{lw}^{HB}. See Supplementary Note 3.

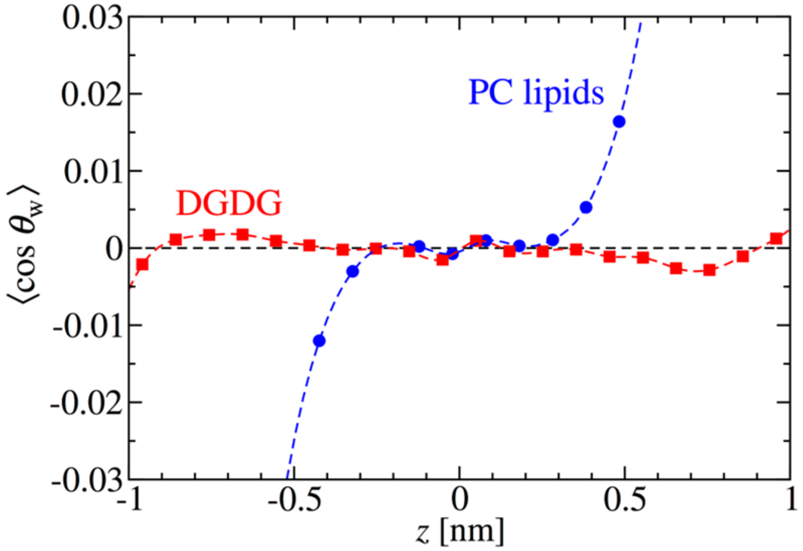

Supplementary Figure 4. **Origin of the long-range repulsion.** Water orientation profiles between DGDG and PC lipid membranes for $D_{\rm w}$ = 2.3 nm. Same as Fig. 5b in the main text but zoomed into the z-range around the centre of the water layer. See Supplementary Note 4.

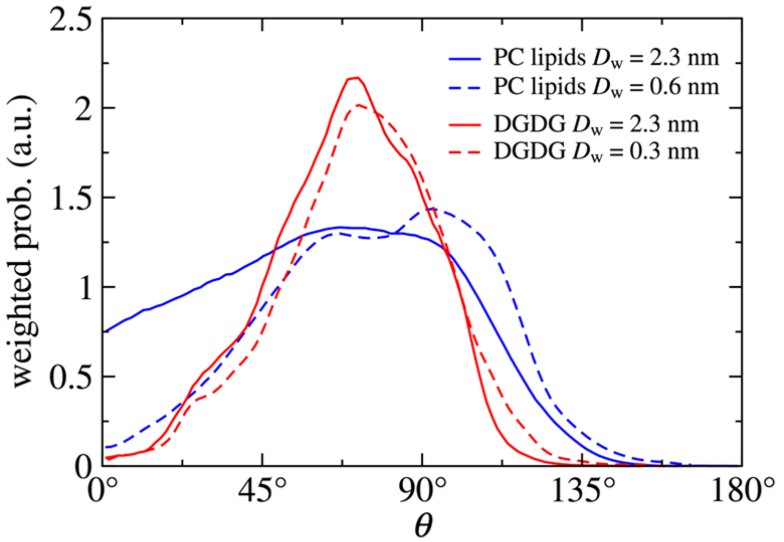

Supplementary Figure 5. **Headgroup orientations.** Angular distribution of DGDG and PC lipid headgroups for small ($\Pi \approx 0$, $D_w = 2.3$ nm) and large ($\Pi \approx 600$ bar, $D_w = 0.6$ nm and 0.3 nm) equivalent interaction pressures. See Supplementary Note 5.

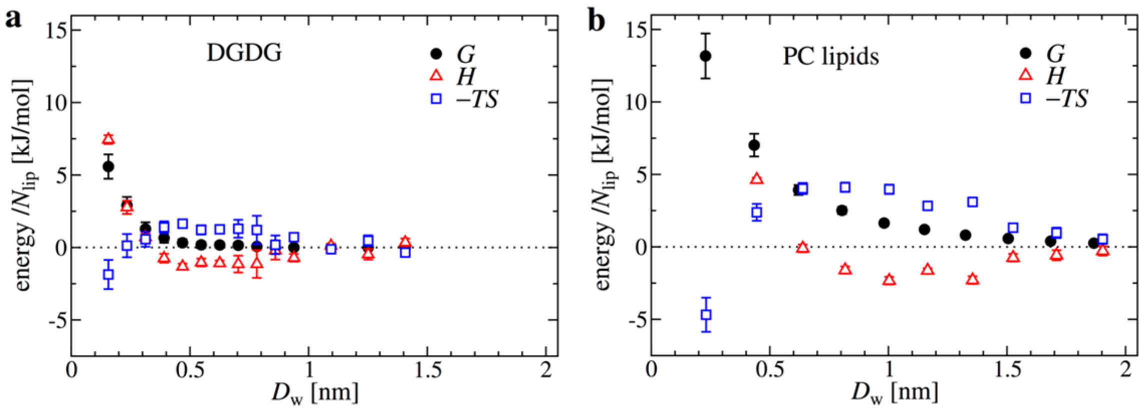

Supplementary Figure 6. **Interaction energetics.** (a) Decomposition of the free energy for DGDG into the enthalpic and entropic contributions. (b) The same free energy decomposition for PC lipids. See Supplementary Note 6.

Supplementary Note 1: Thermal expansion of the area per DGDG lipid

The thermal expansion coefficient of the DGDG bilayer was determined by performing simulations at three different temperatures: 280 K, 300 K, and 320 K. The lengths of the simulations were between 200 and 600 ns and the error associated with the area per lipid was estimated by block averaging. The resulting area per lipid is shown in Supplementary Fig. 1. From the linear fit (dashed straight line) we obtain the thermal expansion coefficient of the area per lipid.

$$\alpha_A = \frac{1}{A_l}\left(\frac{\partial A_l}{dT}\right)_p = 1.1(3) \times 10^{-3}\ \mathrm{K}^{-1}. \quad (1)$$

Supplementary Note 2: Sensitivity to the simulation parameters

The force field used for DGDG is based on GROMOS 53a6 [1], which was developed in combination with the Simple Point Charge (SPC) water model, a Lennard-Jones (LJ) cutoff distance of r_{LJ} = 1.4 nm and a Coulomb interaction modified by a reaction field (RF) contribution. In order to be consistent with the PC lipid membrane simulations and with previous studies [2, 3], we instead use the SPC/E water model, Particle-Mesh-Ewald (PME) electrostatics, and r_{LJ} = 0.9 nm. In the following, we examine the sensitivity of the DGDG area per lipid upon the force field variations mentioned above. Figure 2a in the main text shows the area per DGDG averaged over simulation times of around 80 ns. In addition, we performed longer simulations of 1 μs, for which the results are shown in Supplementary Fig. 2 by red open square symbols. In these additional simulations we estimated the errors associated with the area per lipid by the block averaging method. As seen, the obtained error bars are comparable to those from the main text where they were estimated by the scatter of data points. To determine the effect of variations in the simulation parameters, we first extended the LJ cutoff distance to r_{LJ} = 1.4 nm, which results in the values represented by green open circles. The area per lipid in this case decreases a bit, which can be explained by a longer-ranged lipid–lipid attraction due to the larger LJ cutoff distance. Nevertheless, in both cases, the simulation data points lie close to the experimental values (orange open squares), albeit slightly above in the former and slightly below in the latter case. Further, switching from SPC/E to SPC water and from PME to RF electrostatics (blue triangles) does not influence the final results significantly. The good match between simulated and experimental area per lipid and the fact that the force-field-related differences in the final results seen above are small suggest that the above-mentioned force-field modifications are acceptable. In fact, it was shown that minor variations in the area per lipid have negligible influence on the membrane interaction forces [3].

Supplementary Note 3: Hydrogen bonds in hydrated PC lipid membranes

In the same way as in Fig. 4a in the main text for DGDG, we show in Supplementary Fig. 3 the change in Δn_{tot}^{HB} upon dehydration for PC lipids. Also for PC lipids Δn_{tot}^{HB} increases upon swelling, that is, with D_W. It reaches a saturation value at much larger separations than in the DGDG case. Fitting an exponential curve to the points gives a decay length of λ = 0.43 nm. The fit is, however, not as good as for DGDG in the main text. The inset shows the decomposition of the total number of HBs into water–water (ww) and lipid–water (lw) contributions. Note that the lipid–lipid (ll) contribution is

zero, as PC lipids possess only proton acceptors and therefore cannot form HBs among themselves. The exponential fits (dashed lines) with decay lengths λ = 0.36 nm (ww) and λ = 0.33 nm (lw) match the data points very well.

Supplementary Note 4: Water orientation profiles within the water layer

As shown in Fig. 5b in the main text, the degree of water orientation is much higher in hydrated PC lipid membranes than in hydrated DGDG membranes for the same water layer thickness. In Supplementary Fig. 4, which shows a close-up view of the water orientation within the water slab, it can be seen that for PC lipid membranes significant orientation extends virtually all the way to the centre of the water layer, where it has to vanish by symmetry. For DGDG, on the other hand, orientation within the water layer is almost immeasurably weak.

Supplementary Note 5: Head group angular distributions compared at the same pressure

In Fig. 5c in the main text we compare the angular distributions of DGDG and PC lipid headgroups with respect to the membrane normal at same separations, that is, D_w = 2.3 nm and 0.6 nm. Here, Supplementary Fig. 5 shows a similar comparison but at the same equivalent interaction pressures of $\Pi \approx 0$ and $\Pi \approx 600$ bar, respectively. For the former condition we choose the separation D_W = 2.3 nm, whereas the second one corresponds to D_w = 0.3 nm and 0.6 nm for DGDG and PC lipids, respectively. Even when bringing the DGDG membranes down to a 0.3 nm separation, the impact on the angular distribution is still much weaker than for PC lipids at 0.6 nm.

Supplementary Note 6: Decomposition of the interaction free energy

It can be instructive to decompose the membrane interaction into its enthalpic and entropic contributions. For this purpose, we first quantify the interaction free energy $G(D_w) = A\int \Pi dD_w$, which we can also evaluate via water chemical potential μ and the number of water molecules in the water layer N_W as:

$$\frac{G}{N_{lip}} = \frac{1}{N_{lip}} \int_{N_w}^{\infty} \left(\mu(N'_w) - \mu_0\right) dN'_w \,. \quad (2)$$

In the next step, we evaluate the enthalpy H of the system in dependence of D_w, where we account also for the enthalpy of the water molecules transferred into the bulk

$$H(D_w) = H_0(D_w) - N_w(D_w) h_w \,, \quad (3)$$

where H_0 is the total enthalpy of the simulation box and h_w = −38.9038 kJ/mol is the enthalpy per water molecule in bulk, evaluated from independent bulk water MD simulations. The entropic contribution of the interaction, $-TS$, then follows as $-TS = G-H$. Supplementary Fig. 6a shows G, H, and $-TS$ as functions of D_w for DGDG membranes. Consistent with the rapidly decaying interaction pressure, G is seen to be immeasurably small for D_w > 0.6 nm. This does not apply, however, to H and

$-TS$, which assume magnitudes of 1–1.5 kJ/mol/lipid up to $D_w \approx 0.9$ nm, but are of opposite sign and exhibit virtually complete cancellation. The significant repulsion ($dG/dD_w < 0$) for $D_w < 0.6$ nm is driven by the enthalpy, $dH/dD_w < 0$. In contrast, the entropic contribution is attractive, $-TdS/ dD_w > 0$, but sub-dominant. For $D_w > 0.6$ nm the situation is opposite ($dH/dD_w > 0$ and $-TdS/dD_w < 0$). For a comparison with the commonly studied PC lipid membranes, Supplementary Fig. 6b shows the same enthalpy–entropy decomposition for PC lipids. The overall picture is qualitatively similar for DGDG and PC lipids. In both cases we notice enthalpic repulsion dominating over entropic attraction at low hydration and a turnover to the opposite situation at larger hydration. However, since for PC lipids the entropic repulsion significantly dominates over the enthalpic attraction at large hydration, G for PC lipids assumes substantially positive values in a much wider hydration range, measurable even for $D_w > 1.5$ nm. This substantial entropic repulsion at higher hydration has been discussed previously [2, 4].

Supplementary References

[1] Oostenbrink, C., Villa, A., Mark, A. E. & Van Gunsteren, W. F. A biomolecular force field based on the free enthalpy of hydration and solvation: The GROMOS force-field parameter sets 53A5 and 53A6. *J. Comput. Chem.* 25, 1656–1676 (2004).

[2] Schneck, E., Sedlmeier, F. & Netz, R. R. Hydration repulsion between biomembranes results from an interplay of dehydration and depolarization. *Proc. Natl. Acad. Sci.* 109, 14405–14409 (2012).

[3] Kanduč, M., Schneck, E. & Netz, R. R. Hydration Interaction between Phospholipid Membranes: Insight into Different Measurement Ensembles from Atomistic Molecular Dynamics Simulations. *Langmuir* 29, 9126–9137 (2013).

[4] Markova, N., Sparr, E., Wadsö, L. & Wennerström, H. A Calorimetric Study of Phospholipid Hydration. Simultaneous Monitoring of Enthalpy and Free Energy. *J. Phys. Chem. B* 104, 8053–8060 (2000).
